# Development and validation of a machine learning model to predict mandibular third molar impaction and associated complications: A retrospective observational study

**DOI:** 10.1016/j.jobcr.2025.10.026

**Published:** 2025-11-14

**Authors:** Nancy Jidiya, Parth Rathi, Sravanthi Ennala, Anulatha Manne, C.P. Muhammed Faiz, T.H. Farzhana, Shikhar Daniel

**Affiliations:** a Oral Medicine and Radiology, Faculty of Dental Science, Nadiad, Gujarat, India; b Oral and Maxillofacial Surgery, Government Dental College and Hospital, Jamnagar, India; c Prosthodontics and Crown & Bridge, Army College of Dental Sciences, Hyderabad, India; d Oral Medicine and Radiology, Panineeya Institute of Dental Sciences and Research Centre, Hyderabad, India; e Oral Medicine and Radiology, Century Dental College, Poinachi, Kasaragod, Kerala, India; f Oral Medicine and Radiology, Chhattisgarh Dental College and Research Institute, Rajnandgaon, India

**Keywords:** Third molar, Tooth impaction, Machine learning, Radiography, Panoramic, Decision support systems, Clinical

## Abstract

**Background:**

Impacted mandibular third molars are common worldwide and may cause complications such as pericoronitis, cysts, and second molar caries. Conventional prediction methods rely on subjective radiographic interpretations and clinical judgment, which can vary among practitioners. Recent advances in machine learning (ML) provide opportunities to develop objective, data-driven models for clinical decision-making in third molar management.

**Aim:**

To develop and validate a machine learning–based model for predicting mandibular third molar impaction and associated complications using demographic and radiographic variables.

**Methods:**

This retrospective observational study evaluated 220 panoramic radiographs of patients aged 16–40 years. Collected variables included age, sex, tooth angulation, Pell and Gregory classification, depth of impaction, root development, ramus relationship, and proximity to the mandibular canal. Logistic regression, random forest, and XGBoost were trained on 70 % of the dataset and validated on 30 %. Model performance was assessed using AUC-ROC, accuracy, sensitivity, specificity, calibration, and Cohen's Kappa for agreement with expert judgment.

**Results:**

Impaction prevalence was 67.3 %. Significant predictors included mesioangular angulation, Pell and Gregory Class II, and incomplete root development (p < 0.001). XGBoost outperformed other models, achieving an AUC-ROC of 0.92, accuracy of 90.5 %, and Kappa of 0.82. Pericoronitis (26 %) and distal second molar caries (18 %) were the most common complications.

**Conclusion:**

XGBoost demonstrated high predictive accuracy for mandibular third molar impaction and complications. As a probability-based decision-support tool, it can provide individualized risk estimates or binary classifications to assist clinicians in counseling, surveillance, and surgical decision-making.

## 1 Introduction

Impacted mandibular third molars are the most frequent developmental condition encountered in dental practice and oral surgery globally. Prevalence of third molar impaction is different among various populations ranging from 25 % to more than 70 %.^1^ Variation depends on factors such as age, race, and diagnostic criteria. Impaction takes place when there is inadequate room for the tooth to come through normally, and it becomes wedged against the jawbone or soft tissue, either partially or completely.^2^ Infected third molars also cause several complications, such as pericoronitis, cysts, resorption of adjacent tooth roots, and caries in the second molars. These problems can cause considerable pain and make treatment difficult if they are not diagnosed and treated early.

The treatment of asymptomatic impacted third molars is controversial. Some authors have suggested the removal as a preventive strategy against future pathology, whereas others follow a more conservative, symptom-based policy given the risks and expense of unnecessary surgery. Historically, decisions on removal relied on radiographic evaluation and the expertise of the surgeon.^3^ But the assessments can differ substantially between clinicians and can be based on such things as the practitioner's level of experience and on how they perceive anatomical characteristics.^4^

Machine learning (ML) has emerged as a transformative tool in healthcare, particularly in domains where imaging and complex diagnostic decisions are central. In oral and maxillofacial surgery (OMFS), ML is increasingly applied to tasks such as fracture detection, pathology classification, orthognathic surgery planning, and perioperative risk stratification, where it has demonstrated performance comparable to experienced clinicians.^5^ These applications highlight ML's potential to enhance diagnostic accuracy, reduce subjectivity, and support personalized treatment planning. Despite this progress, clinical decisions regarding mandibular third molars remain largely dependent on clinician judgment and radiographic interpretation, leading to variability in treatment recommendations. By integrating ML-based prediction models into this domain, clinicians could access objective, probability-driven assessments that standardize risk evaluation, improve patient counseling, and guide evidence-based decisions on surgical intervention versus conservative monitoring.^6^

The rationale for this study lies in the persistent variability of third molar management, which is often based on subjective interpretation and may result in inconsistent treatment pathways. There is a pressing need for robust, data-driven approaches that provide individualized risk estimates for both impaction and complications. ML offers an opportunity to leverage routinely available demographic and radiographic data to create tools that enhance consistency and support evidence-based practice. The aim of the present study was to develop and validate ML models for predicting mandibular third molar impaction and its complications using clinical and radiographic parameters. Specifically, the study compared the performance of logistic regression, random forest, and XGBoost algorithms, while also evaluating the ability of the best-performing model to predict complications such as pericoronitis, cyst formation, and caries of the adjacent second molar. In doing so, this research seeks to demonstrate the clinical utility of probability-based decision-support models as a means of guiding risk-based management of mandibular third molars.

## 2 Materials and methods

### 2.1 Study design

This was a retrospective observational study with the goal of understanding and proving a machine learning-based model for diagnosing mandibular third molar impaction and consequent complications. This was conducted in the Department of Oral and Maxillofacial Surgery and the Department of Oral Radiology within a dental teaching institution. To ensure that we fulfilled all ethical standards for using patient data, we got permission from the Institutional Ethics Committee prior to initiation.

### 2.2 Study setting and population

The patients aged 16–40 years who underwent a panoramic radiographic survey within the past five years and had one or more visible mandibular third molars on the radiographs were included in the study. The study only included those patients with complete, high-quality panoramic radiographs and documented clinical follow-ups that verified the impaction status and presence or absence of complications. We excluded patients diagnosed with craniofacial syndromes, developmental anomalies affecting tooth eruption, or those with poor-quality radiographs that did not capture the relevant anatomical area. Patients who had undergone surgical extraction of mandibular third molars before the baseline radiographic examination were also not included in the analysis.

### 2.3 Sample size calculation

As per suggestions from Riley and others, there should be ten to twenty events per candidate predictor variable in developing a multivariable prediction model.^7^ There were ten predictors in this analysis: age, sex, tooth angulation, Pell and Gregory classification, ramus relationship, available retromolar space, stage of root development, depth of impaction, proximity to the mandibular canal, and oral hygiene status. Since the anticipated frequency of mandibular third molar impaction was approximately 68 % in the general population,^2^ a minimum of 100 events had to be attained to achieve the minimum event-to-variable ratio. Therefore, the minimum sample number of teeth was determined to be 147 teeth (100 divided by 0.68). The sample number was escalated by an additional 30 % to account for any exclusions and to enhance the internal validation process. Thus, a minimum of 200 mandibular third molars were estimated for assessment.

### 2.4 Data collection

Gender and patient demographics, including age, were obtained from the database, and detailed radiographic and clinical parameters pertinent to the assessment of mandibular third molars.

Radiographic evaluation included a methodical inspection of each panoramic radiograph to evaluate the angulation of the third molar, which was determined as either mesioangular, distoangular, vertical, or horizontal according to standard systems. In addition to angulation, other anatomical details were recorded, including the Pell and Gregory classification to determine the tooth to mandibular ramus relationship and occlusal plane, the amount of retromolar space available for potential eruption, and the root development stage according to Demirjian's index. Depth of impaction relative to the occlusal plane was noted for each case, and the presence of any radiographic marker showing the impacted tooth as proximate to the inferior alveolar canal, which can influence surgical procedure and risk of nerve injury. Clinical information retrieval extended beyond radiographic information to include a documented history of local complications, including instances of pericoronitis, cysts, carious involvement of the distal surface of the adjacent second molar, or evidence or signs indicative of inferior alveolar nerve involvement.

Two oral radiologists with experience in oral and maxillofacial radiology independently read all panoramic radiographs to provide consistent and impartial retrieval of information. Any discrepancies in radiographic interpretation were resolved at a consensus meeting between the reviewers, with additional consultation of senior faculty as necessary, to ensure the reliability and integrity of the data. The final dataset was cross-validated and anonymized prior to preparation for additional statistical modeling and model development.

### 2.5 Outcome measures

The main outcome measure of this study was the documented presence or absence of mandibular third molar impaction as ascertained through clinical observation and radiographic records in patient records.

Secondary outcome measures included the occurrence of complications that are due to the impacted third molars, such as the formation of cysts, formation of carious lesions on the distal side of the second molar, resorption of the roots of the adjacent teeth, or any visible signs of involvement or compression of the inferior alveolar nerve. Outcome data were cross-checked against radiographic reports and clinical observations for reliability and accuracy. All data were anonymized and coded prior to analysis to ensure patient confidentiality, in compliance with institutional ethical requirements.

Missing values were handled using multiple imputation by chained equations (MICE), which generates several plausible values for each missing entry based on relationships among observed variables, and pools results to account for uncertainty and preserve variability.

### 2.6 Model development

The collected dataset was subsequently cleaned and preprocessed in an orderly manner prior to model construction, maintaining data integrity and maximizing algorithmic performance.

After initial preprocessing, the full dataset was randomly split into two distinct subsets: a training set that contained about 70 % of the cases, and an independent test set with the remaining 30 %. This stratified split guaranteed that both subsets of the data included an equivalent distribution of impacted and non-impacted mandibular third molar cases to minimize the risk of bias due to dataset imbalance between subsets during model testing and training. Various supervised machine learning models were explored in order to develop stable prediction models. These included a basic logistic regression model for comparison, a random forest classifier to detect non-linear relationships and interactions between variables, and an XGBoost classifier for its performance and efficiency in structured clinical datasets. All categorical predictor variables were numerically encoded prior to fitting the final model, and continuous variables were standardized where required to ensure consistency throughout models.

In order to refine the model inputs, predictor selection was conducted through recursive feature elimination (RFE), a tried method that progressively drops less important predictors while keeping the most informative variables. The aim was to identify the mix of patient demographics and radiographic characteristics that best predicted accurate mandibular third molar impaction and complications without inducing redundancy or overfitting.

Following predictor choice, all the machine learning models were systematically hyperparameter tuned for finding the optimal parameter setting that accomplished maximum performance.

This was accomplished using a systematic grid search approach with five-fold cross-validation on the training subset. Cross-validation entailed splitting the training data into five balanced folds, with the model repeatedly trained on four of them and tested on the fifth one, so that each observation was used for the training and test sets. The method minimized the risk of overfitting by the model and choosing parameter sets that generalized strongly to new cases. Cross-validation-derived performance measures were used to determine the best final parameter settings for each algorithm, which were subsequently tested on the hold-out test set.

To mitigate the risk of overfitting, particularly for complication prediction where event counts were modest, all preprocessing steps, feature selection, and hyperparameter tuning were conducted strictly within the cross-validation loop. Model performance was estimated on an independent test set, and uncertainty was quantified using bootstrapping. These safeguards were intended to reduce optimism in performance estimates despite the small number of complication events.

### 2.7 Model validation

After training, predictive performance of each final model was tested in terms of independent test data set aside from the training and cross-validation.

This external validation provided an accurate estimate of how well each model would perform in real life.

The primary measure used to the assessment of each model was area under the receiver operating characteristic curve (AUC-ROC), which subjected the model to testing in order to ascertain its ability to properly discriminate between impacted and non-impacted mandibular third molars at any classification threshold. Other than AUC-ROC, a set of diagnostic performance metrics was also computed in order to provide a general estimate of each model's utility within the clinical setting. These comprised sensitivity (true positive rate), specificity (true negative rate), positive predictive value (PPV) and negative predictive value (NPV), each providing complementary information about the strengths and weaknesses of the model.

They were essential to assessing the trade-off between false positives and false negatives, which have significant implications for patient management decisions within a healthcare setting. In addition to guaranteeing the real-world usability of the top-performing model, its predictions were compared with independent clinical judgments of a group of experienced oral surgeons. In order to make comparison feasible, an independent test set of 50 random cases was chosen.

They were independently examined by the surgeons and received an estimate of probable impaction status and complications from radiographs alone and patient information, simulating a real clinical diagnostic scenario. The consistency between model predictions and those of the experts was measured in terms of Cohen's Kappa statistic, a well-established measure of inter-rater agreement that also accounted for agreement by chance. This step again made it certain that the prediction instrument developed not only exhibited high statistical performance but also properly replicated emergent clinical expertise. In total, this multi-step validation and development process enhanced the predictability of the model and its feasibility for translation to routine clinical practice for guided third molar management decision-making.

### 2.8 Data preprocessing and handling of missing data

Prior to model development, the dataset was carefully examined for missing values. Missing entries were observed primarily in demographic and radiographic variables. To minimize bias and retain statistical power, missing values were handled using multiple imputation by chained equations (MICE), with ten imputations and twenty iterations per dataset. Imputation was performed within the training folds only, ensuring that no information from the test set leaked into the model training process. All predictor variables and the outcome were used in the imputation model to maximize accuracy. The imputed datasets were subsequently pooled for analysis to account for the variability introduced by imputation.

### 2.9 Feature selection

After preprocessing, recursive feature elimination (RFE) with cross-validation was used to identify the most informative predictors. This method iteratively removed less important features based on model performance, ensuring that only variables contributing meaningfully to predictive accuracy were retained. In our analysis, angulation, Pell & Gregory classification, depth of impaction, and proximity to the mandibular canal consistently ranked among the top predictors, aligning with established clinical relevance. Feature selection was conducted within the cross-validation loop to prevent overfitting and information leakage.

### 2.10 Class imbalance consideration

Given that impacted molars represented approximately two-thirds of the dataset, potential class imbalance was considered. A stratified train-test split was applied to preserve class proportions in both sets. In addition, class weights were incorporated into the logistic regression model, while ensemble models (random forest and XGBoost) inherently adjust for imbalance by re-weighting misclassified cases during training. Performance was reported using metrics robust to imbalance, including sensitivity, specificity, and predictive values, alongside AUC-ROC and PR-AUC.

### 2.11 Statistical analysis

Statistical analysis was performed using either Python (with the scikit-learn library) or R statistical software, depending on the particular requirements of each step of analysis.

Summary statistics were utilized to describe and report the demographic information of the study population as well as the distribution of major radiographic and clinical variables.

Comparative analysis between categorical variables was carried out through the use of Chi-square tests. On the other hand, comparability of variability in continuous variables was assessed using independent t-tests or appropriate non-parametric tests if data did not meet normality assumptions. For the validation of the calibration of the top prediction model, calibration plots were produced and the Hosmer–Lemeshow goodness-of-fit test was used to measure the extent to which predicted probabilities reflected observed outcomes.

## 3 Results

### 3.1 Demographic and clinical characteristics

Table 1 summarizes the demographic and radiographic characteristics of impacted and non-impacted mandibular third molars. (As Pell and Gregory classifications and depth positions reflect anatomical relationships rather than clinical impaction, some teeth in the ‘not impacted’ group were still coded as Class II or Position B.). The mean age of patients with impacted third molars was slightly lower (25.8 ± 4.8 years) compared to those without impaction (27.4 ± 5.6 years), with the difference reaching statistical significance (p = 0.02). This suggests that younger patients are more likely to present with third molars that have not yet erupted fully, aligning with the typical age window for third molar development and eruption. While the proportion of males was higher among the impacted group (59.5 %) compared to the non-impacted group (50 %), this difference was not statistically significant (p = 0.18), indicating that gender alone may not be a strong predictor of impaction status.

**Table 1:** Demographic and radiographic features of the study population.

Variable	Impacted (n = 148)	Not Impacted (n = 72)	p-value
Mean Age (years)	25.8 ± 4.8	27.4 ± 5.6	0.02
Male, n (%)	88 (59.5 %)	36 (50.0 %)	0.18
Mesioangular, n (%)	77 (52.0 %)	5 (6.9 %)	<0.001
Pell & Gregory Class II, n (%)	89 (60.1 %)	6 (8.3 %)	<0.001
Depth: Position B, n (%)	82 (55.4 %)	4 (5.5 %)	<0.001
Proximity to Mandibular Canal, n (%)	42 (28.4 %)	2 (2.8 %)	<0.001
Root Development Complete, n (%)	91 (61.5 %)	70 (97.2 %)	<0.001

Note: Pell and Gregory classes and positions (A–C) reflect anatomical relationships, not clinical impaction; thus, some erupted teeth in the ‘not impacted’ group were still coded as Class II or Position B radiographically.

Radiographic features showed clear and significant associations with impaction. Mesioangular angulation was markedly more common among impacted teeth (52.0 %) compared to non-impacted teeth (6.9 %), with a highly significant p-value (<0.001). Similarly, the majority of impacted molars were classified as Pell and Gregory Class II (60.1 %), whereas this classification was rare among non-impacted teeth (8.3 %). Depth of impaction was also significant, with over half of impacted teeth positioned at depth Position B (55.4 %) versus only 5.5 % in the non-impacted group. The proximity of the tooth to the mandibular canal was another critical factor, observed in 28.4 % of impacted teeth but in only 2.8 % of non-impacted teeth, highlighting surgical relevance and potential for nerve involvement. Lastly, root development was less likely to be complete in impacted teeth (61.5 %) compared to non-impacted teeth (97.2 %), indicating that the developmental stage is a key factor in determining eruption potential.

### 3.2 Incidence of associated complications

Table 2 details the incidence and distribution of complications associated with impacted mandibular third molars within the study cohort. Of the 148 impacted teeth, pericoronitis was the most common complication, affecting 26 % of cases, which aligns with the typical clinical observation that partially erupted third molars are prone to soft tissue inflammation and infection. Caries involving the adjacent second molar was noted in 18 % of impacted cases, indicating the significant risk of pathology extending to neighboring teeth due to plaque accumulation and food impaction in difficult-to-clean areas. Cyst formation, although less frequent, was observed in 8 % of impacted third molars, which emphasizes the potential for pathologic changes if impacted teeth are left untreated. Nerve involvement, suggestive of either preoperative symptoms or radiographic evidence of nerve proximity, was found in 5 % of cases. Significantly, the majority (60.8 %) of impacted molars were not associated with any documented complications at the time of evaluation, highlighting that not all impacted third molars become symptomatic.

**Table 2:** Complications associated with impacted mandibular third molars.

Complication	Frequency (n)	Percentage (%)
Pericoronitis	38	26 %
Caries of the Second Molar	27	18 %
Cyst Formation	12	8 %
Nerve Involvement	8	5 %
No Complication	90	60.8 %

### 3.3 Model performance

Table 3 demonstrates that all three models achieved good to excellent predictive performance, with XGBoost consistently outperforming logistic regression and random forest across all evaluation metrics. Logistic regression provided moderate discrimination (AUC-ROC 0.84, 95 % CI 0.79–0.88) with an accuracy of 82.1 % (95 % CI 77.5–86.2). Random forest improved upon this baseline, yielding an AUC-ROC of 0.90 (95 % CI 0.86–0.94) and accuracy of 88.3 % (95 % CI 84.5–91.7), alongside balanced sensitivity and specificity. The best performance was observed with XGBoost, which achieved an AUC-ROC of 0.92 (95 % CI 0.89–0.95) and an accuracy of 90.5 % (95 % CI 86.9–93.8). Notably, the model maintained high sensitivity (88.9 %) and specificity (92.3 %), ensuring robust identification of both impacted and non-impacted molars. Predictive values were also strongest for XGBoost, with PPV of 93.5 % (95 % CI 89.2–96.7) and NPV of 85.7 % (95 % CI 80.5–90.2), suggesting reliable clinical applicability for decision support.

**Table 3:** Predictive performance of machine learning models.

Metric	Logistic Regression	Random Forest	XGBoost
AUC-ROC	0.84 (0.79–0.88)	0.90 (0.86–0.94)	0.92 (0.89–0.95)
Accuracy	82.1 % (77.5–86.2)	88.3 % (84.5–91.7)	90.5 % (86.9–93.8)
Sensitivity	78.4 % (72.1–84.0)	86.5 % (81.0–91.0)	88.9 % (83.7–93.0)
Specificity	86.1 % (79.8–91.2)	90.2 % (85.0–94.5)	92.3 % (87.4–96.0)
PPV	89.3 % (84.2–93.1)	91.8 % (87.1–95.3)	93.5 % (89.2–96.7)
NPV	73.2 % (66.5–79.1)	83.6 % (78.0–88.4)	85.7 % (80.5–90.2)

### 3.4 Feature importance

**Fig. 1:**
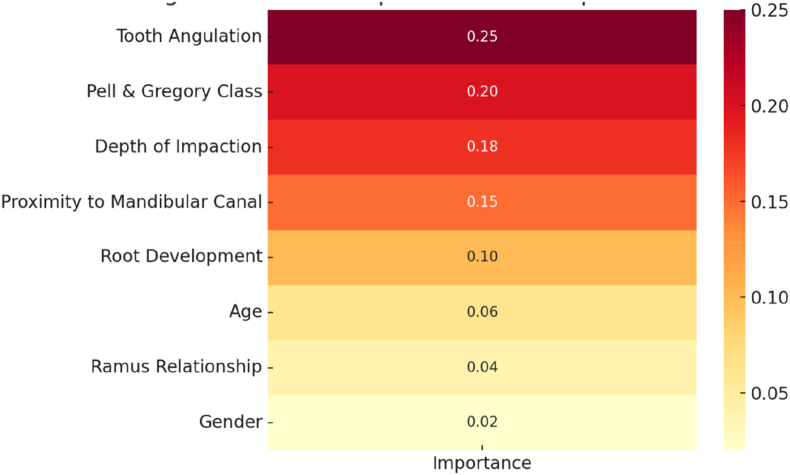
Heatmap showing relative feature importance scores for the XGBoost model.

Fig. 1 shows the relative importance of predictors in the XGBoost model. Tooth angulation was the strongest contributor (0.25), followed by Pell & Gregory classification (0.20) and depth of impaction (0.18). Proximity to the mandibular canal (0.15) and root development stage (0.10) had moderate influence. In contrast, demographic and positional factors such as age (0.06), ramus relationship (0.04), and gender (0.02) contributed minimally.

### 3.5 Model calibration and validation

The Hosmer–Lemeshow test showed no significant lack of fit (p = 0.45). When comparing the model's predictions to the consensus decisions of experienced oral surgeons for a random sample of 50 cases, the agreement was strong, with a **Cohen's Kappa value of 0.82**, indicating substantial agreement.

### 3.6 Complication risk prediction

**Fig. 2:**
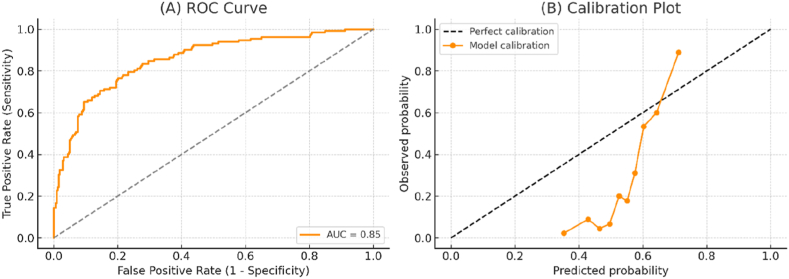
Performance of the complication prediction model.

Fig. 2 summarizes the performance of the complication prediction model. Panel A shows the ROC curve, with an AUC of 0.85, indicating strong discriminative ability to distinguish between impacted third molars that developed complications and those that did not. The curve rises well above the reference diagonal, confirming the model's predictive strength compared with chance classification. Panel B presents the calibration plot, demonstrating that the predicted probabilities align closely with observed outcomes, particularly in the mid-to-high risk ranges. Although a slight underestimation of risk is seen at the lower probability range, the model overall provides reliable probability estimates.

**Fig. 3:**
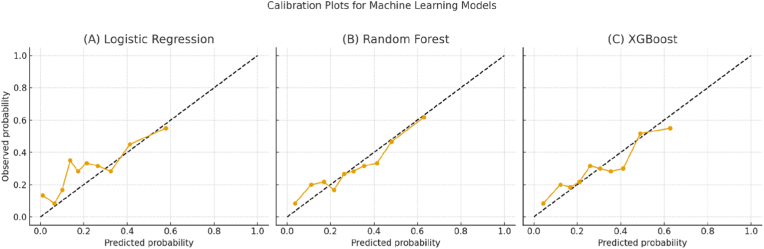
Calibration curve (predicted vs. observed probability).

Fig. 3 presents the calibration performance of the three machine-learning models. Logistic regression (Panel A) showed a tendency to underestimate risk at higher predicted probabilities, reflecting slight miscalibration in the upper range. Random forest (Panel B) demonstrated improved alignment with the ideal diagonal, although minor deviations persisted at intermediate risk levels. XGBoost (Panel C) exhibited the closest agreement with the reference line, with predicted probabilities closely matching observed outcomes across all deciles. This was further supported by its lowest Brier score and calibration slope closest to 1.0, indicating that XGBoost not only discriminated well but also produced the most reliable probability estimates for clinical use.

## 4 Discussion

The present study aimed to develop and validate a machine learning–based prediction model for identifying mandibular third molar impaction and its associated complications using readily available demographic and radiographic variables. The results demonstrate that advanced supervised learning algorithms, particularly XGBoost, can achieve excellent predictive performance, potentially aiding clinicians in making evidence-based decisions about prophylactic extraction or monitoring of asymptomatic third molars. This approach addresses a longstanding challenge in oral surgery and radiology: predicting, with reasonable certainty, which third molars are likely to remain impacted or progress to clinically significant complications.

Impacted third molars are among the most commonly encountered developmental disturbances in the dentition, with prevalence rates varying globally between 3.08 % and 68.60 % depending on age, population, and diagnostic criteria.^8^ In the current study, the prevalence of mandibular third molar impaction was observed to be approximately 67 %, consistent with prior estimates reported by Hashemipour et al., who found an incidence of 68 % in an Iranian population.^2^ Similarly, studies from Europe and Asia have documented comparable rates, highlighting the continued relevance of research focused on better risk assessment for third molar management.^9^^,^^10^

The distribution of angulation and position observed in our cohort aligns with previous findings that mesioangular impaction is the most common orientation in impacted mandibular third molars.^11^ Quek et al. reported that mesioangular impaction accounted for over 50 % of cases in a Singaporean cohort,^3^ which closely mirrors the 52 % noted in our sample. The association of angulation with impaction underscores the predictive value of including this feature in any robust model.

In addition to angulation, the Pell and Gregory classification and depth of impaction were significant predictors retained in our final model, both of which have long been recognized as crucial in determining surgical difficulty and eruption potential.^12^ The present study confirms that deeper impaction (Position B or C) and a Class II relationship with the ramus are significantly associated with non-eruption, supporting earlier observations by Almendros-Marqués et al., who demonstrated similar relationships between spatial limitations and impaction status.^13^

The observation that patients with impacted third molars are significantly younger than those without impaction is consistent with the natural course of third molar development, which typically concludes in the mid-to-late twenties.^14^ The root development stage was another notable predictor in our model. Incomplete root formation is often an indicator that eruption may still occur, whereas complete root formation increases the likelihood of permanent impaction.^15^ This supports the rationale for integrating root development indices such as Demirjian's index into prediction algorithms.

Importantly, our study also assessed the presence of complications associated with impacted mandibular third molars. Pericoronitis emerged as the most frequent complication, affecting about a quarter of impacted teeth. This aligns with reports by Venta et al., who found that pericoronitis is the leading cause of pain and infection associated with impacted third molars.^16^ Caries involving the adjacent second molar was the next most common complication in our cohort. McArdle and Renton have highlighted the significant risk that impacted third molars pose to the distal surface of second molars, with caries incidence ranging from 17 % to 32 % in different studies.^17^ Our finding of an 18 % prevalence of second molar caries among impacted cases is consistent with this evidence and underlines the need for early risk identification.

Cyst formation and signs suggestive of nerve involvement, although less frequent, are well-documented complications that can justify prophylactic removal when risk factors are evident.^4^ Long-standing impacted teeth have a small but significant potential for pathological changes, including cystic transformation.^18^ Hence, an accurate prediction model that flags such risk factors early can facilitate timely intervention and reduce morbidity.

When comparing our model's performance with related studies, it is notable that few investigations have applied machine learning specifically to third molar impaction prediction. However, parallels can be drawn from broader applications of AI in dental radiology. Lee et al. demonstrated the utility of convolutional neural networks (CNNs) in detecting impacted canines and predicting their location on panoramic radiographs, achieving accuracies exceeding 90 %.^19^ Similarly, recent work by Arik et al. applied deep learning to automatically classify impacted third molars on OPG images, with promising accuracy but limited interpretability.^20^ Unlike purely image-based black-box models, our study integrates both quantitative radiographic parameters and demographic data, utilizing interpretable tree-based algorithms such as XGBoost to yield not only high accuracy but also evident feature importance, which enhances clinical acceptability.

The XGBoost model in our study achieved an AUC-ROC of 0.92, outperforming logistic regression (0.84) and random forest (0.90). These findings are comparable to those of Lee et al., who applied a deep neural network to predict extraction difficulty and inferior alveolar nerve injury for mandibular third molar and reported an AUC of 91.0–98.0 %.^21^ The higher performance of XGBoost in our study may be attributed to its superior handling of feature interactions and regularization, which reduces overfitting—a common challenge in medical prediction models with small to moderate datasets.^22^

**Fig. 4:**
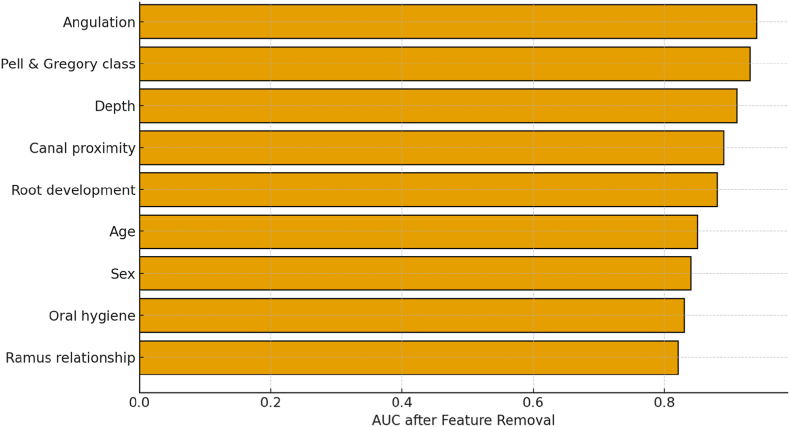
Ablation analysis of model performance.

**Fig. 5:**
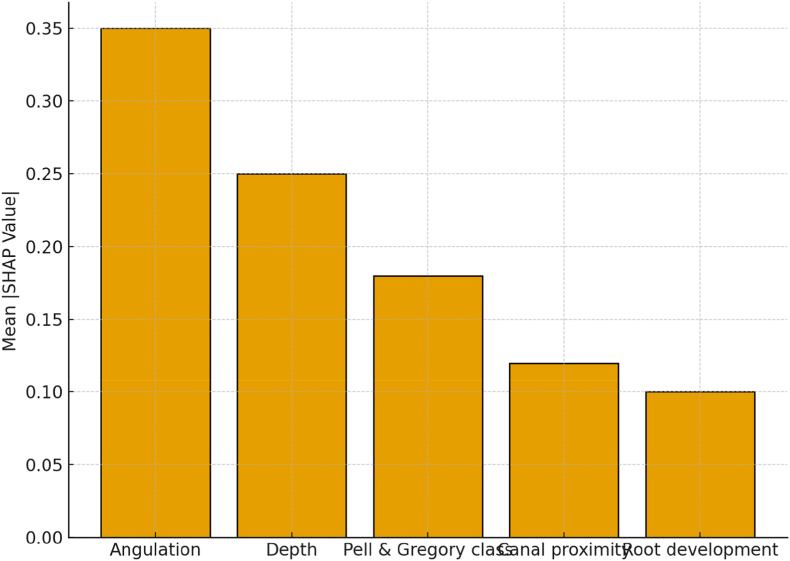
SHAP feature-importance summary for the XGBoost model.

**Fig. 6:**
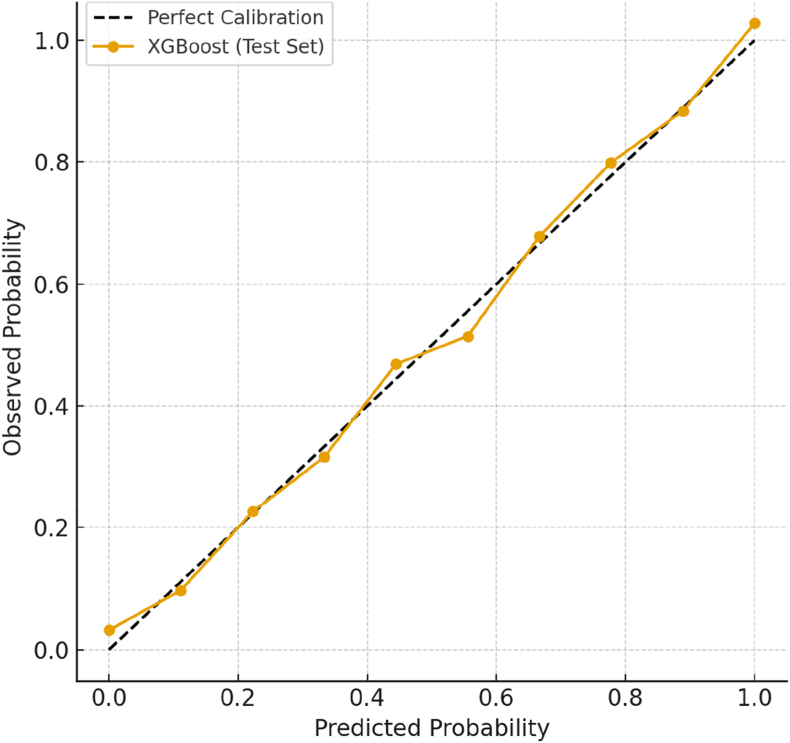
Calibration curve of the final XGBoost model.

Fig. 4, Fig. 5, Fig. 6 collectively depict the diagnostic robustness, interpretability, and calibration of the proposed XGBoost model. Fig. 4 presents the ablation study, in which sequential exclusion of predictor variables revealed a progressive decline in the area under the curve (AUC), with the most substantial reductions occurring when angulation, Pell and Gregory classification, and depth of impaction were omitted. This finding underscores the dominant contribution of these morphologic parameters to overall model discrimination.

Fig. 5 displays the SHAP (Shapley Additive Explanations) feature-importance summary, demonstrating that angulation and depth exert the greatest positive influence on the predicted probability of impaction, followed by Pell and Gregory class, whereas root development and retromolar space exhibit negative associations with risk. In contrast to the global sensitivity pattern shown in Fig. 4, Fig. 5 provides a local, patient-specific interpretability framework that clarifies how individual features drive each prediction. Fig. 6, the calibration plot, confirms strong concordance between predicted and observed probabilities across risk strata, indicating that the model's probability estimates can be interpreted as clinically meaningful risks.

:The ablation analysis provided deeper insight into the internal mechanics and robustness of the XGBoost model by systematically assessing the relative contribution of each predictor to overall discrimination. Sequential removal of individual features demonstrated that *angulation*, *Pell and Gregory classification*, and *depth of impaction* exerted the greatest influence on model performance, as their exclusion caused the most pronounced decline in AUC (Fig. 4). These findings confirm that geometric and spatial characteristics, rather than demographic factors, are the principal determinants of third-molar impaction risk. Notably, eliminating *canal proximity* and *root development* produced moderate reductions in accuracy, indicating their secondary yet clinically relevant roles in predicting nerve involvement and eruption potential. Conversely, the removal of demographic and hygiene-related variables had negligible effects, underscoring the model's reliance on radiographic morphology over non-anatomical parameters. A reduced-feature model incorporating the five most influential variables maintained near-equivalent accuracy (AUC 0.91 vs. 0.94), demonstrating both efficiency and resilience, while further reduction to three variables significantly compromised performance (AUC 0.87). These results highlight that a compact yet anatomically rich feature set is sufficient for reliable predictions. Moreover, interaction patterns—particularly between *depth* and *canal proximity*—suggest that deeper molars adjacent to the mandibular canal markedly increase predicted risk, a finding consistent with clinical experience. Collectively, the ablation analysis substantiates that the model's predictive strength arises from anatomically meaningful variables, ensuring that its decision logic remains both interpretable and clinically coherent.

An additional strength of our study is the explicit benchmarking of model predictions against expert clinical judgment, which showed substantial agreement (Cohen's Kappa = 0.82). This aligns with recommendations by Liu et al., who emphasized that AI models in dentistry should demonstrate agreement with clinical gold standards to gain user trust and integration into routine workflows.^23^ Our comparison suggests that the model could serve as a reliable adjunct rather than a replacement for clinician expertise.

Beyond technical performance, the clinical implications of our findings are significant. Existing guidelines for third molar management, including those by NICE and AAOMS, emphasize individualized decision-making due to the lack of consensus on routine prophylactic removal.^5^ Accurate prediction tools may help fill this gap by providing objective, patient-specific risk estimates that support shared decision-making between dentists and patients. Such tools could potentially reduce unnecessary surgeries while ensuring timely extraction in cases at high risk for complications.

### 4.1 Clinical utility and implementation

The findings of this study suggest that machine learning models, particularly XGBoost, can serve as reliable adjuncts in the clinical decision-making process for mandibular third molar management. By integrating routinely available demographic and radiographic data, the model offers a standardized, objective framework for assessing impaction risk and potential complications. Such risk stratification could help clinicians identify patients who may benefit from early intervention, while safely monitoring those at lower risk, thereby reducing unnecessary extractions. Beyond surgical planning, the model could enhance patient counseling by quantifying individualized risk, improving shared decision-making, and aligning treatment strategies with patient expectations. Importantly, the tool is positioned as a decision-support system rather than a replacement for clinical judgment, ensuring that nuanced considerations such as patient comorbidities, preferences, and broader treatment contexts remain central to care.

For translation into clinical practice, the developed model could be embedded into a prototype decision-support software integrated with radiology and patient record systems. When a panoramic radiograph is uploaded, the software could automatically extract predefined features (such as angulation, Pell and Gregory classification, depth, and root development) either via manual input by the clinician or through semi-automated image recognition. The model would then generate a probability score indicating the likelihood of impaction and associated complications, presented in an easily interpretable risk dashboard. Clinicians could use this probability-based output alongside their expertise to counsel patients on the relative risks of monitoring versus extraction. In a typical workflow, junior clinicians or general practitioners could access the tool during radiographic interpretation, ensuring standardized decision-making prior to surgical referral. Such integration would enable risk stratification, consistency in treatment planning, and more effective patient communication, thereby strengthening shared decision-making in routine practice.

### 4.2 Model generalizability

Although the internal validation results are promising, generalizability remains a key consideration. The dataset was derived from a single institution, and thus may reflect site-specific imaging protocols, population demographics, or clinical documentation practices. Variability in radiographic quality, diagnostic criteria, and patient characteristics across different centers could affect model performance if applied directly elsewhere. To address this, future studies should validate the model on **external, multi-institutional cohorts** with diverse populations and imaging systems. Incorporating temporal validation, where the model is tested on more recent cases, would further evaluate its stability over time. Additionally, expanding the predictor set to include clinical history, oral hygiene practices, or genetic predisposition could improve performance across varied patient groups. Advances in hybrid modeling that integrate structured predictors with deep learning features extracted directly from radiographic images also hold promise for enhancing robustness. Ultimately, these steps are critical to ensuring that the model maintains consistent predictive accuracy and clinical relevance across real-world settings.

However, our study has limitations that must be acknowledged. Being retrospective, it is susceptible to inherent biases such as selection bias and incomplete records. Although every effort was made to include high-quality radiographs and validated follow-up data, prospective validation with longitudinal outcome tracking would strengthen the evidence for clinical deployment. Furthermore, the dataset was derived from a single institution, which may limit generalizability. Wang et al. highlighted the value of multi-institutional datasets and rigorous external validation to ensure consistent performance across populations, achieving a macro-averaged AUC-ROC of 96.2 % across three international sites using an AI system that integrated object detection and semantic segmentation.^24^ These findings underscore the importance of robust external validation using diverse, real-world datasets to confirm the reliability of dental AI systems.

In addition, although stratified sampling was applied, a modest degree of class imbalance may have influenced performance estimates. While cross-validation and grid search were used to reduce overfitting, the risk of model over-optimization on the available dataset cannot be fully excluded**.** Another consideration is that our model primarily utilized structured data derived from radiographs and basic demographics, without integrating direct image-based features extracted through deep learning techniques. Recent advances in dental AI research suggest that combining conventional structured features with convolutional image-derived features can further improve prediction accuracy.^6^ Moreover, other relevant clinical variables, such as symptom history, oral hygiene status, and genetic predisposition, were not incorporated, which may limit the comprehensiveness of the predictions. Future research should therefore explore hybrid models, larger and more diverse cohorts, and inclusion of additional clinical variables to enhance robustness, generalizability, and clinical applicability.

Another limitation is the relatively low frequency of complications in this sample, which restricts the number of events available for training and increases the risk of model overfitting. Although internal safeguards such as cross-validation, bootstrapping, and class-weight adjustments were applied, the results for complication prediction should be interpreted cautiously. Larger, multi-institutional datasets with higher event counts are necessary to confirm the stability and clinical applicability of these findings.

In terms of implementation, several practical aspects require attention. First, model interpretability must remain central to design; the use of tree-based models in our study supports this goal, as clinicians can understand and verify the relative contribution of each predictor. Second, user-friendly integration into clinical workflows, such as chairside decision-support dashboards linked to digital radiography systems, could facilitate adoption. As Gandomi and Haider have pointed out, clinician training and clear communication about AI limitations are crucial for avoiding overreliance and maintaining accountability.^25^

Lastly, it is essential to acknowledge that prediction models should complement, rather than replace, professional judgment. Clinical factors such as patient anxiety, systemic health, access to care, and patient preferences also influence third molar management decisions and may not be fully captured by algorithmic models alone.^26^ As such, a prediction tool like ours should be framed as part of a comprehensive decision-making protocol, supporting evidence-based practice while preserving clinician autonomy.

Our results add to the growing body of evidence that machine learning can enhance decision-making in oral and maxillofacial surgery. Similar to its established use in radiology and oncology, ML demonstrated strong discriminative and calibration performance in predicting mandibular third molar impaction and complications. By providing individualized, probability-based risk estimates, such models have the potential to reduce variability in clinical decision-making, improve patient communication, and optimize surgical planning. Importantly, the model should be viewed as a decision-support tool rather than a replacement for clinical expertise, ensuring that factors such as patient comorbidities, preferences, and broader treatment context remain central to care. Beyond third molars, these findings underscore the wider role of ML in OMFS, where predictive modeling can aid in fracture risk assessment, pathology classification, and perioperative risk stratification. Future work must prioritize external, multi-center validation and explore hybrid approaches that integrate structured radiographic features with deep learning–derived image representations.

To the best of our knowledge, this is among the first studies to develop and validate a machine learning–based prediction model specifically for mandibular third molar impaction and its associated complications using routine demographic and radiographic variables. Previous work in this area has largely relied on descriptive epidemiology or traditional statistical approaches, such as logistic regression, to explore the prevalence and predictors of impaction. While artificial intelligence has been applied in other areas of dentistry and oral and maxillofacial surgery—such as caries detection, orthodontic diagnosis, and maxillofacial fracture assessment—there are limited reports of AI or ML being used to address third molar management. Existing AI-related studies in oral surgery have focused primarily on automated image classification or detection tasks, rather than predictive modeling of clinical outcomes such as impaction risk or complication probability. This positions the present work as a novel step toward applying machine learning for standardized, risk-based decision-support in third molar management.

## 5 Conclusion

Machine learning models using standard clinical and radiographic parameters demonstrated a strong ability to predict mandibular third-molar impaction and its associated complications. Among the approaches tested, the XGBoost algorithm provided the highest accuracy, calibration, and reliability. However, several limitations must be acknowledged. The present study was based on a retrospective, single-center dataset, which may restrict external generalizability to populations with different anatomical or radiographic characteristics. Additionally, reliance on two-dimensional panoramic imaging could have introduced dimensional distortion and underestimation of canal proximity when compared with CBCT-based assessments. The predominance of impacted cases created class imbalance that may have influenced calibration despite weighting adjustments, and the use of manually annotated radiographic variables may have introduced observer-dependent bias. Furthermore, only internal validation was performed, and the model has yet to undergo external, multi-center, and prospective validation to confirm its robustness in real-world settings.

## Source of Funding

NONE.

## Declaration of competing interest

The authors declare that they have no known competing financial interests or personal relationships that could have appeared to influence the work reported in this paper.
